# The relationship between the body mass index and in‐hospital mortality in patients admitted for sudden cardiac death in the United States

**DOI:** 10.1002/clc.23730

**Published:** 2021-11-16

**Authors:** Guy Rozen, Gabby Elbaz‐Greener, Ibrahim Marai, E. Kevin Heist, Jeremy N. Ruskin, Shemy Carasso, Edo Y. Birati, Offer Amir

**Affiliations:** ^1^ Division of Cardiovascular Medicine Hillel Yaffe Medical Center Hadera Israel; ^2^ The Ruth and Bruce Rappaport Faculty of Medicine Technion Haifa Israel; ^3^ Cardiac Arrhythmia Service Massachusetts General Hospital Boston Massachusetts USA; ^4^ Department of Cardiology Hadassah Medical Center Jerusalem Israel; ^5^ Faculty of Medicine Hebrew University of Jerusalem Jerusalem Israel; ^6^ Division of Cardiovascular Medicine Baruch Padeh Medical Center Poriya Israel; ^7^ The Azrieli Faculty of Medicine Bar‐Ilan University Zafed Israel

**Keywords:** BMI, body mass index, obesity paradox, sudden cardiac death

## Abstract

While obesity has been shown to be associated with elevated risk for Sudden Cardiac Death (SCD), studies examining its effect on outcomes in SCD victims have shown conflicting results. We aimed to describe the body mass index (BMI) distribution in a nationwide cohort of patients admitted for an out of hospital SCD (OHSCD), and the relationship between BMI and in‐hospital mortality. We drew data from the U.S. National Inpatient Sample (NIS), to identify cases of OHSCD. Patients were divided into six groups based on their BMI (underweight, normal weight, overweight, obese I, obese II, extremely obese). Socio‐demographic and clinical data were collected, mortality and length of stay were analyzed. Multivariate analysis was performed to identify predictors of mortality. Among a weighted total of 2330 hospitalizations for OHSCD in patients with documented BMI, the mean age was 62.3 ± 29 years, 52.4% were male and 62% were white. The overall rate of in‐hospital mortality was 69.3%. A U‐shaped relationship between the BMI and mortality was documented, as patients with 25 < BMI < 40 exhibited significantly lower mortality (60.7%) compared to the other BMI groups (75.2%), *p* < .001. BMI of 25 kg/m^2^ and below or 40 kg/m^2^ and above, were independent predictors of in‐hospital mortality in a multivariate analysis along with prior history of congestive heart failure and Deyo Comorbidity Index of ≥2. A U‐shaped relationship between the BMI and in‐hospital mortality was documented in patients hospitalized for an out of hospital sudden cardiac death in the United States in the recent years.

## 1 INTRODUCTION

The effect of excess weight on morbidity and mortality has been acknowledged from over 2000 years ago. Hippocrates recognized that “sudden death is more common in those who are naturally fat than in the lean”.^1^ Over the last few decades, the prevalence of obesity in the United States has increased significantly, bearing dramatic social, clinical and economic implications.^2^, ^3^, ^4^, ^5^, ^6^ Elevated body mass index (BMI) has been proven over the years as an independent risk factor for various cardio‐vascular conditions such as ischemic heart disease, acute coronary syndrome, congestive heart failure, atrial and ventricular arrhythmia and sudden cardiac death.^7^, ^8^

Sudden cardiac death (SCD) is responsible for about 50% of the mortality from cardiovascular disease in the United States and other developed countries.^9^, ^10^ Different clinical parameters including age, co‐morbidities, initial cardiac rhythm, and time to return of spontaneous circulation were investigated as predictors of survival in SCD.^11^ While obesity has been shown to be associated with increased incidence and severity of major cardiovascular risk factors and elevated risk for SCD,^8^, ^12^, ^13^ studies examining its effect on outcomes in SCD victims have shown conflicting results.^14^, ^15^, ^16^, ^17^, ^18^, ^19^, ^20^ Some studies showed increased mortality in patients with BMI > 30 kg/m^2^ admitted to the hospital following a sudden cardiac death.^17^, ^18^ At the same time, several other studies have implied that the “obesity paradox”, described in various cardio‐vascular conditions such as acute myocardial infarction (AMI) and heart failure, applies to patients admitted after a sudden cardiac death, showing lower mortality in obese patients.^14^, ^16^, ^18^, ^19^

We aimed at describing the BMI distribution and baseline characteristics in a nationwide cohort of patients, admitted for an out of hospital sudden cardiac death (OHSCD) in the United States, and the relationship between BMI and in‐hospital mortality.

## 2 METHODS

### 2.1 Data source

The data were drawn from the National Inpatient Sample (NIS), the Healthcare Cost and Utilization Project (HCUP), and Agency for Healthcare Research and Quality (AHRQ)^21^, ^22^ datasets, consisting only of de‐identified information; therefore, this study was deemed exempt from institutional review by the Human Research Committee.

The NIS is the largest collection of all‐payer data on inpatient hospitalizations in the United States. The dataset represents an approximate 20% stratified sample of all inpatient discharges from U.S. hospitals.^23^ This information includes patient‐level and hospital‐level factors such as patient demographic characteristics, primary and secondary diagnoses and procedures, co‐morbidities, length of stay (LOS), hospital region, hospital teaching status, hospital bed size, and cost of hospitalization. National estimates can be calculated using the patient‐level and hospital‐level sampling weights that are provided by the HCUP.

For the purpose of this study, we obtained data for the years 2015 (last quarter) and 2016. International Classification of Diseases, 10th Revision, Clinical Modification (ICD‐10‐CM) was used from the last quarter of 2015 and thereafter for reporting diagnoses and procedures in the NIS database during the study period. For each index hospitalization, the database provides a principal discharge diagnosis and a maximum of 29 additional diagnoses, in addition to a maximum of 15 procedures. The reason we only included the data coded with ICD‐10 codes is that the ICD‐10 system includes individual codes for BMI values and ranges.

### 2.2 Study population and variables

We identified patients 18 years of age or older with a primary diagnosis of sudden cardiac death based on ICD‐10‐CM codes I46.2, I46.8, or I46.9, who had one of the BMI, Z68.x, codes, among the secondary diagnoses. Of notice, these represented only the successfully resuscitated OHSCD patients, since those who were not successfully resuscitated in the field or died in the emergency departments, were not hospitalized. To have the “cleanest” possible data on patients admitted for successfully resuscitated out of hospital cardiac arrest, we avoided including patients with a secondary diagnosis of a cardiac arrest in our analysis due to the fact that these could represent patients who underwent an in‐hospital sudden cardiac death or had a prior history of cardiac arrest included as a secondary diagnosis.

The following codes represent the six BMI subgroups we have created for our study: Z68.1, BMI ≤19 kg/m^2^, under‐weight group; Z68.20–25, BMI 20–25 kg/m^2^, normal‐weight group; Z68.26–30, BMI 26–30 kg/m^2^, over‐weight group; Z68.31–35, BMI 31–35 kg/m^2^, obese I group; Z68.36–39 kg/m^2^, BMI 36–39, obese II group; Z68.4, BMI ≥40 kg/m^2^, extremely obese group. In addition to analyzing the individual BMI subgroups mentioned above, we combined the overweigh, Obese I and Obese II groups to compare the outcomes of these patients to the combined group of all the underweight, normal weight and extremely obese patients.

The following patient demographics were collected from the database: age, sex, and race. Prior comorbidities were identified by measures from the AHRQ. For the purposes of calculating Deyo‐Charlson Comorbidity Index (Deyo‐CCI), additional comorbidities were identified from the database using ICD‐10‐CM codes. Deyo‐CCI is a modification of the Charlson Comorbidity Index, containing 17 comorbidity conditions with differential weights, with a total score ranging from 0 to 33. (Detailed information on Deyo‐CCI provided in the Appendix A table). Higher Deyo‐CCI scores indicate a greater burden of comorbid diseases and are associated with mortality, 1 year after admission.^24^ The index has been used extensively in studies from administrative databases, with proved validity in predicting short‐ and long‐term outcomes.^25^, ^26^ Our primary outcome in this study was in‐hospital mortality. Length of stay was the secondary outcome we analyzed.

### 2.3 Statistical analysis

The chi‐square (*χ* ^2^) test and Wilcoxon Rank Sum test were used to compare categorical variables and continuous variables, respectively. The NIS provides discharge sample weights that are calculated within each sampling stratum as the ratio of discharges in the universe to discharges in the sample.^27^ We generated a weighted logistic regression model to identify independent predictors of in‐hospital mortality. Candidate variables included patient‐level characteristics, Deyo‐CCI and hospital‐level factors. We retained all predictor variables that were associated with our primary and secondary outcome with *p* < .05 in our final multivariable regression model. For all analyses, we used SAS® software version 9.4 (SAS Institute Inc., Cary, NC.) A *p* value <.05 was considered statistically significant.

## 3 RESULTS

### 3.1 Study cohort

A total of 466 hospitalizations for successfully resuscitated out of hospital sudden cardiac death patients across the United States during 2015 (last quarter) and 2016 were included in the analysis. After implementing the weighting method, these represented an estimated total of 2330 hospitalizations for OHSCD, in patients with documented BMI during the index hospitalization. The majority of patients (52.4%) were male and the mean age of the cohort was 62.3 ± 29 years.

As shown in Table 1, 62% of the study population were white, majority of 56% had Medicare coverage, 82.3% were in the lower 75th income percentile. As to the clinical characteristics, 39.5% of the study population had history of hypertension, 40.1% had diabetes mellitus, 33.3% had chronic pulmonary disease, 9.9% of the patients had a prior history of an AMI, 10.9% had peripheral vascular disease. The median BMI in the study was 38 (IQR: 31–41) with 85.6% of the patients with BMI above the normal (>25 kg/m^2^). The data reveal that 16.3% of all hospitalizations for successfully resuscitated OHSCD included patients with a diagnosis of VT/VF and 9.2% of the patients were diagnosed with an acute myocardial infraction (STEMI or NSTEMI). Twelve percent of the patients underwent a percutaneous coronary angiography and 2.8% of the patients required percutaneous coronary intervention.

**TABLE 1:** Baseline characteristics of the study population (total and per BMI groups)

	<20	20–25	26–30	31–35	36–39	≥40	Total	*P* Value
Patients, *n*								
Unweighted	42	25	48	74	66	211	466	
Weighted	210	125	240	370	330	1055	2330	
Age group, %								<.001
18–44	9.5	8.0	10.4	8.1	7.6	10.4	9.4	
45–59	23.8	16.0	20.8	21.6	25.8	34.1	27.7	
60–74	31.0	36.0	58.3	52.7	56.1	45.0	47.4	
75 or older	35.7	40.0	10.4	17.6	10.6	10.4	15.5	
Gender, %								<.001
Male	57.1	64.0	56.2	60.8	43.9	48.8	52.4	
Female	42.9	36.0	43.8	37.8	56.1	51.2	47.4	
Missing	0.0	0.0	0.0	1.4	0.0	0.0	0.2	
Race, %								<.001
White	69.0	52.0	47.9	63.5	69.7	63.0	62.4	
Non‐White	31.0	44.0	41.7	16.2	19.7	26.5	26.8	
Other/Missing	0.0	4.0	10.4	20.3	10.6	10.4	10.7	
Comorbidity, %								
Hypertension	26.2	36.0	41.7	50.0	47.0	36.0	39.5	<.001
Congestive heart failure	11.9	12.0	20.8	16.2	27.3	37.0	27.0	<.001
Diabetes Mellitus	9.5	20.0	35.4	55.4	45.5	42.7	40.1	<.001
Renal Failure	19.0	36.0	31.3	33.8	34.8	38.9	34.8	<.001
Peripheral Vascular Disease	14.3	16.0	4.2	12.2	9.1	11.4	10.9	.004
Prior MI	4.8	8.0	18.7	12.2	9.1	8.5	9.9	<.001
VT/VF	9.5	16.0	14.6	27.0	19.7	13.3	16.3	<.001
Deyo‐CCI, %								.008
0	9.5	12.0	14.6	12.2	15.2	10.9	12.0	
1	23.8	16.0	20.8	14.9	13.6	13.7	15.7	
2 or higher	66.7	72.0	64.6	73.0	71.2	75.4	72.3	
Primary payer, %								<.001
Medicare	61.9	72.0	56.3	58.1	53.0	53.1	56.0	
Medicaid	11.9	12.0	14.6	10.8	13.6	19.9	15.9	
Private insurance	11.9	16.0	14.6	23.0	21.2	22.7	20.4	
Self‐pay	7.1	0.0	10.4	4.1	3.0	1.9	3.6	
No charge	2.4	0.0	0.0	0.0	0.0	0.0	0.2	
Other/missing	4.8	0.0	4.2	4.1	9.1	2.4	3.9	
Income percentile, %								<.001
0 to 25th percentile	33.3	52.0	27.1	31.1	31.8	38.9	35.6	
26th to 50th percentile	26.2	12.0	18.8	28.4	34.8	26.1	26.2	
51st to 75th percentile	21.4	12.0	20.8	24.3	18.2	22.7	21.5	
76th to 100th percentile	19.0	20.0	29.2	13.5	12.1	10.9	14.6	
Missing	0.0	4.0	4.2	2.7	3.0	1.4	2.1	
Hospital status, %								<.001
Urban teaching	69.0	80.0	66.7	67.6	80.3	63.0	68.0	
Urban nonteaching	23.8	16.0	31.2	23.0	12.1	28.9	24.7	
Rural	7.1	4.0	2.1	9.5	7.6	8.1	7.3	
Hospital region, %								<.001
South	42.9	40.0	52.1	39.2	47.0	45.0	44.6	
West	23.8	32.0	14.6	17.6	15.2	14.7	17.0	
Midwest	21.4	16.0	18.8	29.7	19.7	28.9	25.3	
Northeast	11.9	12.0	14.6	13.5	18.2	11.4	13.1	
Hospital bed size, %								.159
Large	57.1	52.0	52.1	56.8	48.5	56.9	54.9	
Small/medium	42.9	48.0	47.9	43.2	51.5	43.1	45.1	

*Note*: *p* Values were generated using Chi‐square test and refer to differences between BMI groups within baseline characteristics.

### 3.2 Patients characteristics by BMI group

Baseline characteristics of the study population in the different BMI groups is presented in detail in Table 1. The distribution of the BMI groups varied significantly based on the country regions as well as income percentiles (*p* < .001). While about three quarters of the OHSCD patients (74.9%) across the country were obese (BMI > 30 kg/m^2^), the prevalence of obesity among the study population was highest in the Midwest (81.4%) and lowest in the West coast of the United States (68.3%), *p* < .001. Female predominance was documented in the obese II and the extremely obese groups. Younger age and higher prevalence of comorbidities including hypertension, diabetes and congestive heart failure were documented in the obese patients (Table 1). Among the obese patients, only 12.1% had an annual income in the highest income quartile, compared to 22.7% among nonobese patients (*p* < .001).

### 3.3 Length of stay and mortality by BMI groups

**FIGURE 1:**
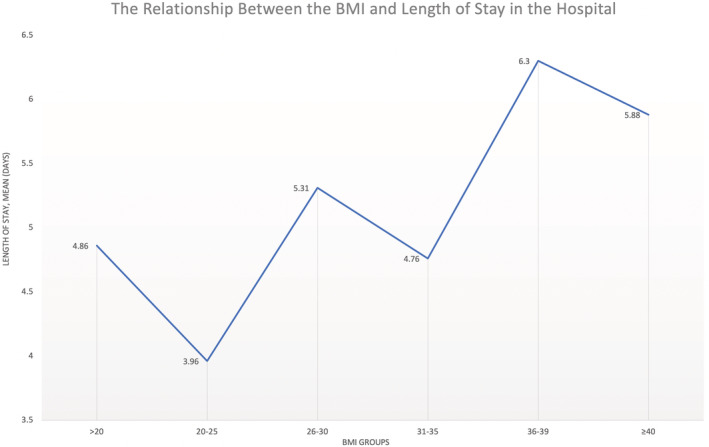
The relationship between BMI and the length of hospitalization in the study

The average LOS in the hospital for the study population was 5.51 ± 0.42 days. As shown in Figure 1; the trend of the correlation between BMI and length of stay was linear in nature with longer hospital stay in obese patients, *p* < .001 (Figure 1).

**FIGURE 2:**
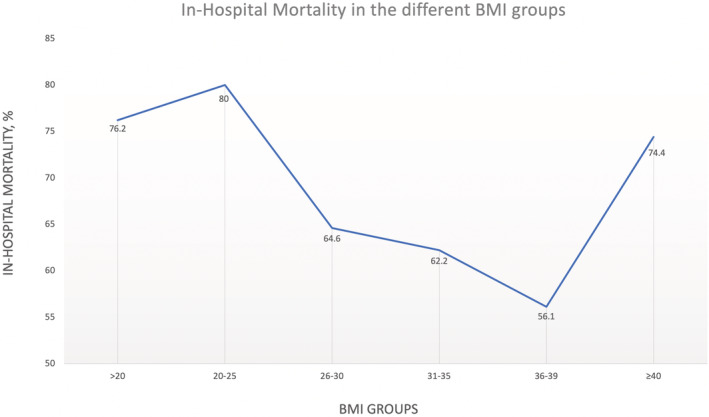
The relationship between BMI and in‐hospital mortality in the study population

The overall rate of in‐hospital mortality during the study period was documented at 69.3%. A U‐shaped relationship between the BMI and the in‐hospital mortality was documented, as described in Figure 2. Following the observation that the over‐weight, obese I and obese II patient subgroups (BMI 26–39) exhibited significantly lower in‐hospital mortality (61%) compared to the other BMI groups (75%), we performed an additional statistical analysis dividing the patients into these two subgroups (Table 2).

**TABLE 2:** In‐hospital outcomes for the total study population and per BMI group

BMI, kg/m^2^	≤20	20–25	26–30	31–35	36–39	≥40	Total	*p* Value
Mortality, %	76.2	80.0	64.6	62.2	56.1	74.4	69.3	<.0001
Length of Stay (days), Mean ± SEM	4.86 ± 0.85	3.96 ± 0.41	5.31 ± 0.27	4.76 ± 0.58	6.30 ± 0.57	5.88 ± 0.56	5.51 ± 0.42	<.0001

*Note*: *p* Values were generated using Chi‐square test and refer to differences between BMI groups.

### 3.4 Predictors of in‐hospital mortality

In an unadjusted analysis, we found several parameters that significantly increased the odds of in‐hospital mortality (Table 3). These included: white race and history of congestive heart failure (all with *p* < .01). In addition, BMI 26–30, BMI 31–35, BMI 36–39 (compared to BMI 20–25) and having BMI between 26 and 39 compared to the other groups combined (below 25 kg/m^2^ or above 40 kg/m^2^), decreased the risk for in‐hospital mortality (all with *p* < .01). Personal history of hypertension and diabetes were found as predictors of improved outcomes in a univariate analysis, before adjusting for potential confounders. While admission with a diagnosis of STEMI or NSTEMI did not predict improved outcomes, the small proportion of patients who underwent coronary intervention (2.8%) were found to have lower mortality, Odds Ratio (OR) –0.21, 95% Confidence Interval (95% CI), (0.17–0.28). After adjusting for potential confounders, BMI below 25 kg/m^2^ or above 40 kg/m^2^, compared to BMI 26–39 kg/m^2^, remained an independent predictor of in‐hospital mortality in a multivariate analysis (Table 4). Hypertension and diabetes were not found to be independent predictors in a multivariate analysis, implying an interaction between them and other clinical parameters directly effecting the primary outcome. Congestive heart failure, OR −1.29 (1.01–1.65), and Deyo Comorbidity index of ≥2, OR −1.64 (1.19–2.25), were also found to be independent predictors of mortality.

**TABLE 3:** Univariate analysis for predictors of in‐hospital mortality

Predictor	Probability (95% CI)	Odds Ratio (95% CI)	*p* Value
Age group, years			**.487**
18–44	72.73% (66.47,78.20)	1.00 (reference)	N/A
45–59	70.54% (66.91,73.93)	0.90 (0.64,1.26)	.537
60–74	68.33% (65.52,71.00)	0.81 (0.59,1.12)	.198
75 or older	68.06% (63.06,72.67)	0.80 (0.55,1.16)	.235
Race			**.008**
Non‐White	65.60% (61.79,69.22)	1.00 (reference)	N/A
White	71.48% (69.10,73.74)	1.31 (1.08,1.61)	0.008
BMI Sub‐groups			**<.001**
Below 20	76.19% (69.96,81.47)	0.80 (0.47,1.37)	.419
20–25	80.00% (72.07,86.11)	1.00 (reference)	N/A
26–30	64.58% (58.33,70.38)	0.46 (0.27,0.76)	.003
31–35	62.16% (57.11,66.96)	0.41 (0.25,0.67)	<.001
36–39	56.06% (50.66,61.33)	0.32 (0.20,0.52)	<.001
40 and above	74.41% (71.69,76.95)	0.73 (0.46,1.15)	.174
BMI 2 groups			**<.001**
25–39	61.02% (57.92,64.04)	1.00 (reference)	**N/A**
Below 25 or 40 and above	75.28% (72.91,77.50)	1.94 (1.63,2.32)	**<.001**
Gender			**.172**
Male	70.49% (67.87,72.99)	1.00 (reference)	N/A
Female	67.87% (65.06,70.56)	0.88 (0.74,1.05)	.172
Hospital bed size			**<.001**
Large	72.27% (69.75,74.65)	1.00 (reference)	N/A
Small/medium	65.71% (62.79,68.53)	0.74 (0.62,0.88)	<.001
Diabetes mellitus			**.010**
No	71.33% (68.90,73.64)	1.00 (reference)	N/A
Yes	66.31% (63.22,69.27)	0.79 (0.66,0.95)	.010
Hypertension			**.011**
No	71.28% (68.86,73.58)	1.00 (reference)	N/A
Yes	66.30% (63.19,69.29)	0.79 (0.66,0.95)	.011
Congestive heart failure			**.004**
No	67.65% (65.38,69.83)	1.00 (reference)	N/A
Yes	73.81% (70.23,77.09)	1.35 (1.10,1.65)	.004
Deyo‐CCI			**.075**
0	64.29% (58.50,69.68)	1.00 (reference)	N/A
1	72.60% (67.80,76.93)	1.47 (1.05,2.06)	.024
2 or higher	69.44% (67.19,71.59)	1.26 (0.97,1.65)	.086
*Clinical course*			
PCI			**<.001**
No	70.20% (68.28,72.05)	1.00 (reference)	N/A
Yes	38.46% (27.49,50.74)	0.27 (0.16,0.44)	<.001

Abbreviations: CI, confidence interval; Deyo‐CCI, Deyo Comorbidity Index.

*Note*: *p* Values in bold refer to the global null hypothesis of no difference between the subgroups. *p* Values not in bold refer to the pairwise comparison of each subgroup with the reference subgroup.

**TABLE 4:** Multivariate analysis for predictors of in‐hospital mortality

Predictor	Probability (95% CI)	Odds ratio (95% CI)	*p* Value
Age group, years			**.078**
18–44	72.05% (64.03,78.86)	1.00 (reference)	N/A
45–59	63.50% (56.80,69.72)	0.68 (0.46,1.00)	.050
60–74	64.08% (58.03,69.70)	0.69 (0.47,1.02)	.061
75 or older	59.41% (52.04,66.39)	0.57 (0.37,0.87)	.010
Gender			**.325**
Male	66.04% (60.54,71.14)	1.00 (reference)	N/A
Female	63.76% (58.07,69.09)	0.90 (0.74,1.10)	.325
Race			**.002**
Non‐White	60.68% (53.94,67.04)	1.00 (reference)	N/A
White	68.92% (64.33,73.16)	1.44 (1.15,1.80)	.002
BMI sub‐groups			**<.001**
Below 20	72.62% (64.82,79.24)	0.84 (0.48,1.47)	.546
20–25	75.88% (65.77,83.74)	1.00 (reference)	N/A
26–30	61.64% (53.40,69.26)	0.51 (0.30,0.88)	.016
31–35	52.20% (44.46,59.84)	0.35 (0.21,0.58)	<.001
36–39	54.16% (46.19,61.91)	0.38 (0.22,0.64)	<.001
40 and above	69.84% (64.67,74.55)	0.74 (0.45,1.20)	.218
BMI 2 groups			**<.001**
25–39	56.04% (50.17,61.74)	1.00 (reference)	N/A
Below 25 or 40 and above	71.48% (66.82,75.73)	1.97 (1.61,2.40)	<.001
Hospital bed size			**<.001**
Large	68.86% (63.60,73.68)	1.00 (reference)	N/A
Small/medium	60.74% (54.90,66.29)	0.70 (0.57,0.85)	<.001
Deyo‐CCI			**.003**
0	56.02% (47.81,63.92)	1.00 (reference)	N/A
1	70.46% (63.48,76.59)	1.87 (1.27,2.75)	0.001
2 or higher	67.57% (62.81,71.99)	1.64 (1.19,2.25)	0.003
Congestive heart failure			**.043**
No	64.35% (59.21,69.18)	1.00 (reference)	N/A
Yes	69.93% (63.04,76.03)	1.29 (1.01,1.65)	.043
Percutaneous coronary intervention			**<.001**
No	65.34% (60.28,70.08)	1.00 (reference)	N/A
Yes	33.50% (20.98,48.88)	0.27 (0.15,0.49)	<.001

Abbreviations: CI, confidence interval; Deyo‐CCI, Deyo Comorbidity Index.

*Note*: *p* Values in bold refer to the global null hypothesis of no difference between the subgroups. *p* Values not in bold refer to the pairwise comparison of each subgroup with the reference subgroup.

## 4 DISCUSSION

Utilizing data from the NIS, the largest all‐payer inpatient database in the United States, we analyzed a weighted total of 2330 hospitalizations between October 2015 and December 2016, after an out of hospital sudden cardiac death. This nationwide data analysis documented a U‐shaped relationship between the BMI and in‐hospital mortality in OHSCD patients hospitalized in the United States during the study period. BMI between 26 and 39 m/kg^2^ was found to be independent predictor of lower in‐hospital mortality, in patients hospitalized for a successfully resuscitated OHSCD.

Some of the prior studies, investigating the relationship between BMI and survival after a sudden cardiac death, analyzed specific patient subgroups like post MI sudden cardiac death patients^28^ or patients treated with therapeutic hypothermia.^17^, ^18^ Other studies, analyzing larger populations, investigated long‐term outcomes of patients who survived hospitalization following an out of hospital cardiac arrest,^14^ or in‐hospital outcomes in large cohorts of patients who underwent resuscitation for in‐hospital cardiac arrest only.^16^, ^19^ In a recent meta‐analysis, Ma et al. combined seven prospective and retrospective studies, involving 25 035, including out of hospital and in‐hospital sudden cardiac death patients.^29^ The patients were divided into four BMI groups: underweight (BMI < 18.5), normal weight (18.5 ≤ BMI < 25), overweight (25 ≤ BMI < 30) and obese (BMI ≥ 30) and the meta‐analysis showed that underweight patients had the worst survival rates compared to normal weight patients (OR 1.35; 95% CI 1.10–1.66; *p* = .004), while overweight patients did better (OR −0.8 [0.65–0.98]; *p* = .03) and obese had similar survival as normal weight patients.^29^

Our goal was to assess the relationship between BMI and in‐hospital course and outcomes in a nationwide, general population of patients, hospitalized following a successfully resuscitated out of hospital cardiac death in the US, overcoming the potential “selection bias” in prior studies. We also aimed to better differentiate the different subgroups of patients suffering from obesity, dividing the obese patients into mild (30 < BMI≤35), moderate (35 < BMI < 40) and extremely obese (BMI ≥ 40) groups.

The clinical characteristics of the patient population in this study were consistent with prior publications on SCD in regard to the median age and different comorbidities such as diabetes mellitus (DM), hypertension (HTN) and history of a myocardial infarction (MI).^14^, ^16^, ^28^, ^30^ The in‐hospital survival to discharge rates, reported in prior studies, ranged between 20.4% and 44%.^15^, ^19^, ^31^ This study documented 30.7% survival to discharge rate in a nationwide population of patients hospitalized after successfully resuscitated OHSCD, within the range of the previously reported outcomes. The above‐mentioned similarities in the patient population characteristics and outcomes provide external validation to our results as we show the relationship between the patients BMI and study outcomes.

Our data revealed a U‐shaped relationship between the BMI and the in‐hospital mortality, while the over‐weight, obese I and obese II patient subgroups (BMI 26–39) exhibited significantly lower in‐hospital mortality. Several other studies have implied that the “obesity paradox”, described in various cardio‐vascular conditions such as acute myocardial infarction and heart failure, applies to patients admitted after a sudden cardiac death, showing a lower mortality in obese patients.^14^, ^16^, ^18^, ^19^ Importantly, majority of these studies combined all the patients with BMI > 30 in the same study group showing improved outcomes. We subdivided these patients in three groups of mild (30 < BMI ≤ 35), moderate (35 < BMI < 40) and extremely obese (BMI ≥ 40) groups, and showed that only in the first two, the mortality decreased, while extremely obese patients' the mortality was higher.

As shown in Table 1, the overweight and obese patients in our study were younger, a finding that could have contributed to their improved survival. This observation was described previously as well, while 20 out of 26 reports included in a 2014 meta‐analysis of the “obesity paradox” in acute myocardial infarction studies by Niedziela et al., documented younger patients in the overweight and obese populations.^32^

Not surprisingly, patients in the higher BMI groups suffered from increased prevalence of cardiovascular risk factors such as DM and HTN. Some prior studies reported better outcomes in patients with hypertension,^14^, ^18^ while diabetes was associated with worse outcomes post OHSCD.^14^ In our analysis, in a multivariate regression analysis, neither diabetes nor hypertension, were not found to independently predict increased mortality in this nationwide cohort, when corrected for other risk factors including BMI. It is possible that the adverse effect of these comorbidities on patient's outcomes is counterbalanced by the protective mechanisms that improve the survival in obese patients, many suffering from hypertension and diabetes. Several such protective pathophysiological mechanisms were suggested to play a role in improving survival of critically ill patients, including the benefit of nutritional and caloric reserves in obese patients^8^, ^33^, ^34^ and neurohormonal mechanisms related to higher leptin levels in obese patients.^35^, ^36^, ^37^ We assume that similar mechanisms may play a role in patients who are exposed to an intense continuous metabolic stress associated with an OHSCD event and hospitalization.

This association of obesity with favorable survival after OHSCD should not be interpreted as supporting weight gain. Elevated BMI has been proven over the years as an independent risk factor for various cardio‐vascular conditions such as ischemic heart disease, acute coronary syndrome, congestive heart failure, atrial and ventricular arrhythmia and sudden cardiac death.^7^, ^8^ Obese patients suffered their cardiac arrest event years earlier than nonobese patients, providing unequivocal evidence to support preventive strategies to reduce the prevalence of obesity.

Our study should be interpreted in the context of several limitations. First, the NIS database is a retrospective administrative database that contains discharge‐level records and as such is susceptible to coding errors. Second, the lack of patient identifiers in the NIS database prevented us from using other outcome variables and mortality measures such as at 30 days. We could only capture events that occurred in the same index hospitalization. The NIS database also does not include detailed information about patients' clinical characteristics, medication, blood tests etc. Therefore, we cannot rule out residual confounding of the association we observed. These limitations are counterbalanced by the real world, nationwide nature of the data, lack of selection bias as well as absence of reporting bias introduced by selective publication of results from specialized centers.

### 4.1 Conclusion

A U‐shaped relationship between BMI and in‐hospital mortality was documented in patients hospitalized for out of hospital sudden cardiac death in the United States in the recent years. These findings support the existence of an “obesity paradox” in OHSCD, associated with improved in‐hospital survival.

## Data Availability

Data availability statement The data from the national database used for this study will not be made available to other researchers for purposes of reproducing the results or replicating the procedure due to restrictions on the sharing of data in the Healthcare Cost and Utilization Project (HCUP) Data Use Agreement. The National Inpatient Sample (NIS) database is publicly available for purchase and the transparent and detailed methods that are described below make it possible for anyone who wishes to do so to reproduce our results.

## Appendix A.

**Table:**

ICD‐10 CM codes	Condition	Score
I21.x, I22.x, I25.2	Myocardial infarction	1
I09.9, I11.0, I13.0, I13.2, I25.5, I42.0, I42.5–I42.9, I43.x, I50.x, P29.0	Congestive heart failure	1
I70.x, I71.x, I73.1, I73.8, I73.9, I77.1, I79.0, I79.2, K55.1, K55.8, K55.9, Z95.8, Z95.9	Peripheral vascular disease	1
G45.x, G46.x, H34.0, I60.x–I69.x	Cerebrovascular disease	1
F00.x–F03.x, F05.1, G30.x, G31.1	Dementia	1
I27.8, I27.9, J40.x–J47.x, J60.x–J67.x, J68.4, J70.1, J70.3	Chronic pulmonary disease	1
M05.x, M06.x, M31.5, M32.x–M34.x, M35.1, M35.3, M36.0	Rheumatologic disease	1
K25.x–K28.x	Peptic ulcer disease	1
B18.x, K70.0–K70.3, K70.9, K71.3–K71.5, K71.7, K73.x, K74.x, K76.0, K76.2–K76.4, K76.8, K76.9, Z94.4	Mild liver disease	1
E10.0, E10.l, E10.6, E10.8, E10.9, E11.0, E11.1, E11.6, E11.8, E11.9, E12.0, E12.1, E12.6, E12.8, E12.9, E13.0, E13.1, E13.6, E13.8, E13.9, E14.0, E14.1, E14.6, E14.8, E14.9	Diabetes	1
E10.2–E10.5, E10.7, E11.2–E11.5, E11.7, E12.2–E12.5, E12.7, E13.2–E13.5, E13.7, E14.2–E14.5, E14.7	Diabetes with chronic complications	2
G04.1, G11.4, G80.1, G80.2, G81.x, G82.x, G83.0–G83.4, G83.9	Hemiplegia or paraplegia	2
I12.0, I13.1, N03.2–N03.7, N05.2–N05.7, N18.x, N19.x, N25.0, Z49.0–Z49.2, Z94.0, Z99.2	Renal disease	2
C00.x–C26.x, C30.x–C34.x, C37.x–C41.x, C43.x, C45.x–C58.x, C60.x–C76.x, C81.x–C85.x, C88.x, C90.x–C97.x	Any malignancy including leukemia and lymphoma	2
I85.0, I85.9, I86.4, I98.2, K70.4, K71.1, K72.1, K72.9, K76.5, K76.6, K76.7	Moderate or severe liver disease	3
C77.x–C80.x	Metastatic solid tumor	6
B20.x–B22.x, B24.x	Acquired immunodeficiency syndrome (AIDS)	6
